# Detection of Biventricular Volume Increase in Overweight and Obese Individuals Using a Novel Index of the “Standard Human”—A Single-Center, Non-Contrast-Enhanced Cardiac CT Study

**DOI:** 10.3390/jcm15062350

**Published:** 2026-03-19

**Authors:** Maciej Sosnowski, Wojciech Wojakowski, Jan Harpula, Tomasz Lepich

**Affiliations:** 1 Unit of Noninvasive Cardiovascular Diagnostics, 3rd Chair of Cardiology, Faculty of Medical Science in Katowice, Medical University of Silesia, Ziolowa Str 47, 40-635 Katowice, Poland; 2 Department of Cardiology and Structural Heart Disease, 3rd Chair of Cardiology, Faculty of Medical Science in Katowice, Medical University of Silesia, Ziolowa Str 47, 40-635 Katowice, Poland; wwojakowski@sum.edu.pl (W.W.); j.harpula@sum.edu.pl (J.H.); 3 Department of Normal Anatomy, Faculty of Medical Sciences in Katowice, Medical University of Silesia, Ziolowa Str 47, 40-635 Katowice, Poland; lepich@sum.edu.pl

**Keywords:** anthropometry, standard human index, body surface area, cardiomegaly, non-contrast-cardiac computed tomography, biventricular volume, stanines, age

## Abstract

**Background/Objectives**: Biventricular volume (BVV) can be measured from non-contrast-enhanced CT images in patients undergoing coronary artery calcium (CAC) scoring. BVV correlates with left ventricular mass and may predict mortality risk in type 2 diabetes mellitus patients. This study examines the relationships among body size, age, and BVV using the Standard Human Index (SHI), which combines height and bodyprint (BP = k × height − body surface area, h-BSA; k = 1 for females, 1.1 for males). We hypothesize that this novel indexing method enhances the discrimination of increased BVV in overweight and obese patients and assesses the relevance of age in interpreting BVV changes. **Methods**: We analyzed CT data from 2466 patients (1606 women, 860 men; mean age 64 ± 11 years) referred for CAC scoring. Fatless BVV was measured semi-automatically, and we compared raw BVV values and BVV normalized for height, body surface area (BSA), and the SHI across sex, age, and body mass index (BMI) categories. **Results:** BVV was significantly higher in males (414 ± 97 mL) than females (297 ± 66 mL) (*p* < 0.001). BVV decreased non-linearly with age, stabilizing in older patients. Normal-weight males had higher BVV than females (*p* < 0.001). Normalization for height, BSA, and the SHI indicated that BSA did not effectively distinguish BVV changes in overweight and obese patients. **Conclusions:** The proposed index effectively diagnosed BVV increases in overweight individuals, while BSA indexing may be misleading. The age dependence of BVV challenges the validity of standards based on younger populations for detecting ventricular enlargement in older adults.

## 1. Introduction

Cardiomegaly, an increase in cardiac size, is traditionally viewed as a sign of heart damage linked to various diseases, including non-cardiac conditions. It can be recognized as a pattern or measured as an increase in overall cardiac volume, which may involve the enlargement of the atria, ventricles, or both [1,2]. Standard ranges for normal heart size often focus on specific cardiac chambers, neglecting overall heart size, and limits for normality have not been established [3]. Clinically, it is important to note that heart enlargement may not be detectable in many disease states; conversely, a normal-sized heart does not rule out disease, like in aortic stenosis, hypertrophic cardiomyopathy, and electrical heart disease [4,5,6,7]. Importantly, a smaller heart may be linked to circulatory incompetence and fatal outcomes in certain conditions [8,9,10,11].

The data indicate that the increase in atrial and ventricular volumes does not carry equal risk for life-threatening cardiovascular complications. An enlarged left atrium (LA) has been shown as not independently associated with overall mortality [12,13], irrespective of the LV diastolic function. The opposite was also reported: LA volume (LAV) determined on routine cine-CMR was independently associated with all-cause mortality, but missed the LV diastolic function [14]. The increase in ventricular size in patients with heart failure has been proven as associated with the mortality rate as high as 50% over 5 years [15]. This distinction is crucial as detecting ventricular enlargement, here described by BVV, regardless of ejection fraction, poses a greater challenge in preventing sudden or premature death than detecting atrial enlargement [16].

Coronary artery calcium (CAC) scoring has been integrated into clinical practice over the last four decades for the early detection of atherosclerosis and is recognized as a valuable coronary risk modifier [17,18,19]. This examination involves minimal exposure to X-ray radiation and does not require contrast media, allowing for the assessment of various anatomical structures, including the right and left atria separately and the cardiac ventricles together with muscle mass [20,21]. However, in non-contrast cardiac CT, it was impossible to distinguish between the two ventricular chambers and their walls. The earliest attempts date back 30 years [22,23], and for a long time, the bi-ventricular volume assessment was the only approach, but it has not been widely applied [24,25]. Further clinical evidence of the practical value of the biventricular volume determination in type 2 diabetes has been provided [26,27]. Given that the number of patients undergoing CAC testing worldwide can reach tens of millions and is constantly growing, determining additional indicators, among which heart size, particularly, is of life-threatening importance, in a large population may be feasible without incurring extra biological and procedural costs.

Absolute and normative values often prove ineffective for assessing heart size in individual patients. For decades, researchers have sought optimal methods to index parameters describing the size of cardiac chambers, as well as the mass and volume of large thoracic vessels, to minimize inter-individual variation [28,29]. Current recommendations typically advise indexing ventricular and atrial volumetric parameters to body surface area (BSA) [3]. For left ventricular mass (LVM), height raised to the power of 2.7 is the recommended denominator [30]. Similarly, guidelines for vascular disease consider height the most appropriate scalar for assessing the size of the thoracic aorta [31]. Interestingly, body mass index (BMI), a common indicator of nutritional status, is seldom considered a normalization factor for heart and vessel size. This is clinically significant in obese or underweight individuals, particularly when cardiovascular disease is present, as the natural progression of such disease often involves cardiac enlargement. The use of these diverse denominators is supported by well-documented prognostic studies that utilize predefined clinical events as endpoints. Nonetheless, disentangling the distinct effects of obesity from those of primary cardiovascular disease remains a considerable clinical and methodological challenge.

To address this, we introduced a new indexing parameter, the Standard Human Index (SHI, unitless), which is a sum of two components: height (unitless) and bodyprint (BP, unitless), defined as the difference between height (unitless) and body surface area (BSA, unitless). The BSA was calculated after the modified Mosteller equation, as [(h × w)/36]^0.5^, where height is expressed in meters and weight in kilograms (further treated as numbers) [32]. In the “ideal human”, taking into account the sexual dimorphism, the k × h = BSA, where “k” equals 1.0 in females and 1.1 in males, and the BP is zero. Any deviation from “zero” indicates the domination of body mass (BP < 0) or height (BP > 0). The SHI normalization has already been examined for total heart volume [33] and left atrial volume [34] and was found to be superior for detecting changes related to body size, providing more accurate assessments than those associated with systemic cardiovascular risk.

Our study aims to track the changes in cardiac biventricular volumes (BVVs) across nine age-strata, using standard nine statistics (stanines) [35] to examine the effects of normalization for BSA, height (H), and the SHI in relation to body mass index categories. We hypothesize that a novel indexation way allows for better discrimination of the increased biventricular volume in overweight and obese patients.

## 2. Materials and Methods

### 2.1. Study Population

Our study was conducted as a single-center, retrospective, observational study of a cohort of 2466 patients who underwent non-contrast-enhanced cardiac computed tomography (NCE-CCT) at our center. The NCE-CCT examination was ordered by the referring cardiologist for the coronary artery calcium (CAC) scoring. These were either symptomless patients with risk factors for coronary artery disease or a positive family history, or low-risk symptomatic patients presenting with atypical or non-anginal chest pain, dyspnea on exertion, unexplained arrhythmias, or when a high CAC score precluded reliable contrast-enhanced coronary angiography.

### 2.2. Cardiovascular Risk Factors Evaluation

**Figure 1:**
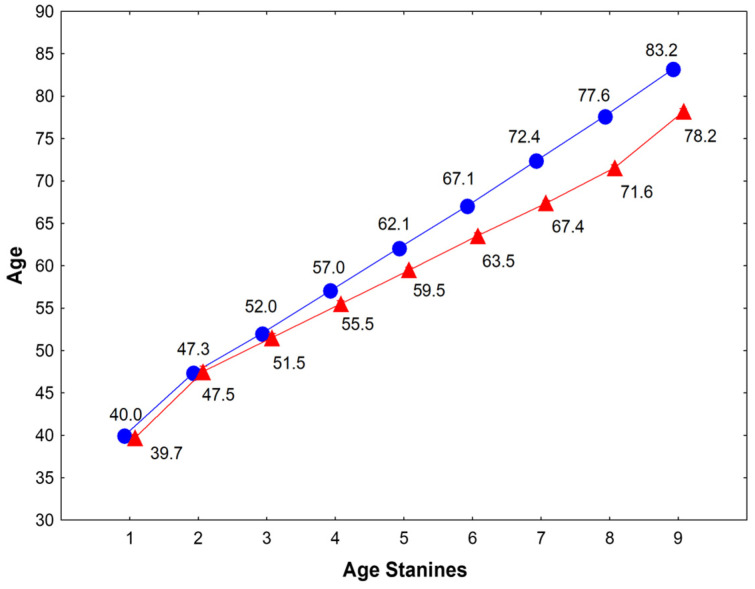
Means of age-stanines in women (blue circle) and men (red triangles). The different values exemplify longer life-expectancy in women (data population-specific, not generalizable). [Stanines follow as: 1st < M −1.75 SD (mean and standard deviation), 2nd M −1.75 to −1.25, 3rd M −1.25 to −0.75, 4th M −0.75 to −0.25, 5th M ± 0.25 SD, 6th M 0.25 to 0.75, 7th M 0.75 to 1.25, 8th M 1.25 to 1.75, 9th > M + 1.75 SD.].

In each subject, age (years), sex (women/men), height (h, meters), weight (W, kilograms), body mass index (BMI, kg/m^2^), smoking habits, systemic arterial blood pressure, lipids level, and diabetic state were noticed according to a simple risk assessment protocol (Table 1 and Table 2). Nine age-related categories (stanines) were calculated in women and men separately on the basis of their age distribution across the whole population in Poland (see Section 2.6, Figure 1) [35]. Normal weight (BMI < 25), overweight (BMI 25–29.9), and obese (BMI ≥ 30) categories were used. Smoking habits were categorized as ever- (past or current) and never-smoking. Systemic arterial hypertension (SAH) has been reported in individuals who were previously diagnosed with hypertension, were taking antihypertensive medication, had blood pressure measured before a CT scan equal to or greater than 180/100 mmHg, or required urgent lowering of blood pressure. Dyslipidemia (HL) was classified in subjects treated with lipid-lowering agents or who had documented total cholesterol (TC) of 190 mg/dL or more, or low-density lipoprotein cholesterol (LDL) of 100 mg/dL or more, or a triglyceride (TG) concentration of 150 mg/dL or more, or both.

Type 2 diabetes mellitus (T2DM), previously diagnosed, was recorded only if patients were treated with insulin and/or oral medications. Patients with abnormal oral glucose tolerance tests or elevated fasting glucose levels, as well as those on an antidiabetic diet, were classified as nondiabetic unless they were receiving treatment. There were no patients with type 1 diabetes mellitus.

### 2.3. Coronary Artery Calcium Determination and Risk Assessment

Coronary artery calcium (CAC) examinations were performed without the use of a contrast agent (non-contrast-enhanced, NCE-CCT) using a 64-row multi-detector CT (Aquilion, Toshiba, Japan) or 2 × 192-row (SOMATOM Force Dual Source, Siemens Healthcare GmbH, Forchheim, Germany) with predefined prospective scanning adjusted to heart rate in mid-diastolic phase (MD, ~60–80% of the cardiac cycle duration). Multi-planar (2D and 3D) offline reconstructions of the images were performed on Vitrea-2 workstations (software v. 3.9.0.0. Vital Images, Minnetonka, MN, USA, or Syngo.via (v. VB30A_HF06). The coronary calcification burden was assessed manually or semi-automatically following the method of Agatston et al. [17]. A commercially available standardized reporting format for CACS was used. Coronary artery calcium score was added to classic risk factors to categorize cardiovascular risk following conditions: CACS < 10 Agatston units, normotensive, and non-diabetic as low-risk; and CACS ≥ 10 or hypertension or type 2 diabetes or smoker and high lipids or structural abnormalities (a posteriori) as high-risk.

### 2.4. Bi-Ventricular Size Evaluation

**Figure 2:**
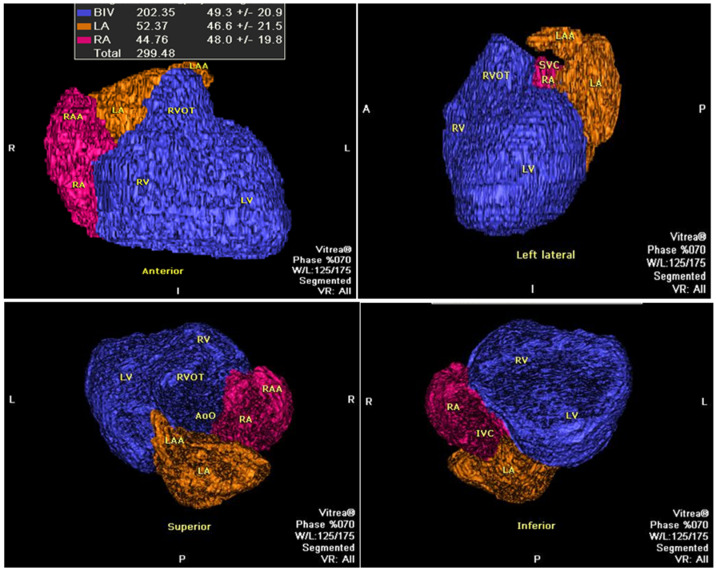
An example of the determination of fat-free cardiac volume. Abbreviations: BIV—bi-ventricular, IVC—inferior caval vein, LA—left atrium, LAA—left atrial appendage, LV—left ventricle, RA—right atrium, RAA—right atrial appendage, SVC—superior caval vein, RVOT—right ventricular outflow tract. The BVV (=BIV) in blue is a whole presentation of left and right ventricles’ blood volumes, ventricular mass (muscle), and the outflow trunk.

To estimate the biventricular volume (BVV), we utilized commercially available software on the Vitrea-2 workstation, specifically employing the “general” module and the “organ” function. This software facilitated a semi-automatic determination of the volume of designated anatomical structures using a 3D object detection technique based on radiological density. Manual corrections were performed in nearly all cases to ensure accuracy. To address potential inaccuracies, we reviewed the volume rendering of the entire heart region and selectively isolated the ventricular components. Any inappropriate volume rendering (VR) areas were manually adjusted or removed to ensure that only the relevant ventricular volumes were included in the final analysis. Notably, epicardial fat areas, with averaged Hounsfield units around −70 ± 30, were excluded from the analysis. The mid-diastolic (MD) biventricular volume (BVV) was defined as the combined volume of both ventricles, including ventricular mass (muscle) and volume (blood), as well as the outflow tracts, which cannot be differentiated using non-contrast-enhanced cardiac computed tomography (Figure 2).

### 2.5. Measurements Reproducibility

**Figure 3:**
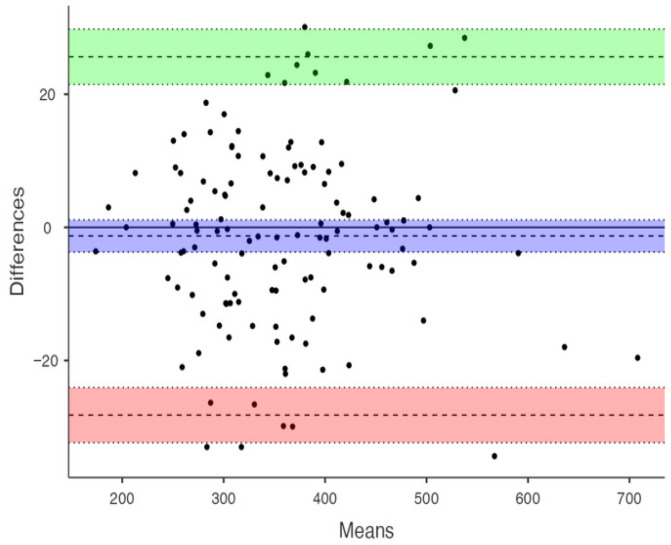
Bland–Altman plot of the BVV measurements reproducibility X-axis: means, (BVV1 + BVV2)/2, [mL]); Y-axis: difference, BVV1 − BVV2 [mL]. The purple bar represents estimated bias ± 95% CI; the green and pink bars represent upper and lower limits of agreement (LOA).

All measurements were performed by an experienced investigator (MS, >7K manually corrected measurements, which provided >12K individual parameters of the cardiac chambers, including BVV). The intra-observer reproducibility of the measurements was examined in 126 randomly selected subjects by the investigator (MS), made from 6 weeks to 2 years apart from the first evaluation. The concordance correlation coefficients of repeated measurements with reached CCC = 0.988. The absolute bias was −1.28 [95CI −3.70, 1.14], the upper and lower limits of agreement were 25.64 [95CI 25.64–29.79] and −28.20 95CI −32.35–24.50 m (Figure 3).

### 2.6. Statistical Analysis

Quantitative data are presented as means ± 1 standard deviation or median and interquartile range, depending on their distribution. Qualitative data are presented as numbers or proportions. Student *t*-test was used to compare normally distributed parametric data. Distributions were quantified using the chi-square test. The general regression model was used to analyze the dependency of BVV indexed for body size metrics on anthropometric and clinical factors. Age-strata were calculated for women and men using 9-standards (Stanine) statistics. It divides a distribution of scores into nine equal parts, or “stanines,” ranging from 1 to 9. Each stanine represents a range of scores, with stanine 5 being the average or mean score [35]. Reference data have been allocated for the 2010 year statistics of longevity in Poland [36]. For reproducibility analysis, correlation analysis, and the mean errors were calculated. Statistical packages were used (Statistica 8.0. StatSoft Inc. Tulsa, OK, USA, and R statistics v.4.5.2., The R Foundation for Statistical Computing, 2025). A *p*-value < 0.05 was considered significant.

### 2.7. Ethics

The study was conducted in accordance with the Declaration of Helsinki [37]. The analysis was performed on a dataset comprising solely non-identifiable variables: height, weight, sex, and age. As no direct or indirect identifiers were present, this research qualified for exemption from an ethics committee review under institutional and national guidelines for anonymized research. Also, the Ethics Committee of the Medical University of Silesia ruled that consent is not required for retrospective studies. Regardless, patients were required to provide informed consent to undergo X-ray examinations at our hospital for diagnostic purposes. They also signed a consent form to share their anonymized data for future, unspecified retrospective analyses.

## 3. Results

### 3.1. Examined Cohort Characteristics

The mean age was similar in BMI-related male subgroups, while the mean age was older in females with overweight and obesity. Height was similar in all comparable subgroups, except for obese women (Table 2). Mean BSA increased in accordance with the BMI classes, with the greatest in the obese women and men, with a clear gender-related difference (Table 2). The novel index of body size, SHI, showed the lower values as the BMI category increased; however, the SHI values in the obese males corresponded with the SHI values in overweight females (Table 2). As the SHI correlated with BMI, and as the bodyprint (BP = h − BSA, unitless) is a critical part of the proposed equation for SHI, proportion of individuals was calculated for normal weight, overweight and obese subgroups in comparison with BP-related subgroups of weight-dominated, height-dominated or with height–weight balance (Table 3).

### 3.2. Biventricular Volumes in the Examined Cohort

The mean biventricular volume was 338 ± 98 mL and it was greater in men than in women by ~100–120 mL, with the means of 297 ± 66 mL and 414 ± 97, resp. The BVV increased with body weight regardless of gender (Table 4), with statistically significant marginal, although statistically significant, increase between normal-weight and overweight women and a greater increase in obese women (Table 4). A more gradual increase was observed in men (Table 4).

Mean BVV was higher in women with a weight predominance and in men with a weight–height balance. Lower values were observed in both sexes with height predominance. Use of height and the SHI as denominators showed concordance, whereas use of the BSA resulted in averaging BVV values in 5 of the 6 categories (Table 5).

### 3.3. Biventricular Volumes and Age-Stanines

**Figure 4:**
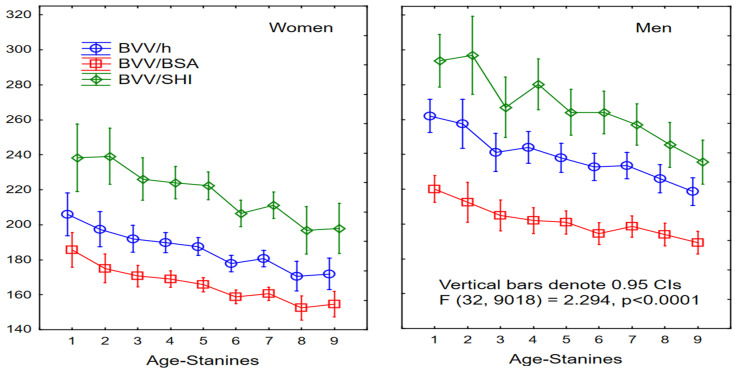
Age-related normalized BVV changes. BVV—biventricular volume (mL); h—height; BSA—body surface area, after Mosteller [32]; SHI—standard human index; Age-Stanines, gender-related as in Figure 1. ANOVA was used for statistical analysis.

The measured BVV declined with age, being the largest in younger women and men from the 1st age-stanine, with values of 342 ± 106 mL and 467 ± 112 mL, respectively. The mean BVV stabilized from the 3rd age-stanines in both sexes (312 ± 78 mL and 420 ± 79 mL). Then it decreased from the 6th age-stanine (287 ± 60 mL), until the 9th age-stanine with the smallest size of 272 ± 44 mL in women, and from the 8th age-stanine with the smallest size at the 9th age-stanine of 379 ± 90 mL in men. The changes in indexed values for BVV across age-stanines are shown in Figure 4.

### 3.4. Biventricular Volumes and Cardiovascular Risk

The BVV was similar in females irrespective of CV risk. Among males of all BMI categories, BVV was comparable between low- and high-risk (Table 6) individuals. The overall relationships of BVV with age, sex, and risk factors have been estimated regarding the entire cohort (Table 7).

## 4. Discussion

This study presents a methodological and conceptual framework based on the Standard Human Index (SHI), creating a distinct, personalized description of the human body. The research integrates robust clinical data—drawn from a real-world, unselected cohort assessed by certified cardiologists using stringent diagnostic criteria—with technical innovation in cardiac computed tomography (CT) analysis. Going beyond the traditional assessment of coronary artery calcification, biventricular volume (BVV) was included at a specific phase of the cardiac cycle, with accuracy and reproducibility comparable to other methods [24]. The SHI serves as the core analytical advancement, integrating genetic determinants (e.g., height) and regulatory influences (e.g., nutrition, physical activity) into a unified, unitless metric termed the bodyprint (BP), defined as h − BSA. This formulation preserves physical interpretability while avoiding stigmatizing labels, aligning with the conceptual neutrality of established metrics like BMI. Crucially, the SHI correlates strongly with BMI yet provides deeper mechanistic insight into individualized body composition. Clinically, the SHI enables accurate indexing of BVV, particularly where traditional methods failed. In individuals with BP ≈ zero, representing metabolic and morphological balance of weight and height, the BSA or SHI yield consistent results. However, in cases of increased weight and significant bodyprint deviation, conventional BSA-based indexing often normalizes pathologically increased BVV, misclassifying high-BMI patients as “healthy” despite underlying cardiomegaly. The SHI corrects this by accounting for personalized body size physiology, ensuring that cardiac enlargement is detected reliably even in overweight populations. Theoretical rigor underpins the SHI: indexing by height alone is logically valid only under the specific condition of a bodyprint of zero, where BVV/h = BVV/SHI. In all other scenarios, the SHI provides a physiologically coherent denominator that reflects both genetic and regulatory dimensions of body size. By combining rigorous clinical methodology with an innovative body size metric, this work offers a unified framework for the cardiovascular diagnostics’ improvement.

Our results showed a decline in mean biventricular volume with age, which aligns with findings from previous studies [38,39,40]. The trajectories we observed are consistent with data from the Copenhagen General Population Study (CGPS), which reported a decrease in mean BVV across fixed decade cohorts from 439 to 366 mL in men and from 328 to 273 mL in women [38]. This consistency emerged despite a fundamental difference in our methodological approach.

A key innovation of our study is the application of sex-specific age stanines to characterize the aging process in an unselected clinical population. To our knowledge, this method is novel in clinical cardiology and moves beyond the common practice of applying arbitrary, fixed age ranges. Instead, it leverages the intrinsic properties of stanines to tailor the analysis to the specific demographic structure of the cohort, providing a powerful, unbiased tool for precisely tracking physiological changes across the lifespan. This approach yielded a detailed, population-anchored description: in Polish males, mean BVV declined from 467 mL (1st stanine, <45 years) to 379 mL (8th–9th stanines, >75 years), while in Polish females, values declined from 342 mL to 272 mL across the same stanines. A major strength afforded by this method is the robust characterization of older adults; our analysis comprised 755 seniors, compared to only 33 in the ≥70 cohort of the cited CGPS sub-study [38]. In our analysis, we relied on 2010 Polish population data to establish age standards. We recognize that this reliance presents a limitation, as these age standards may not be directly applicable to other ethnic groups. Population data are not collected annually, and year-to-year changes in height and weight values are generally minimal. However, over decades, differences may become more evident. The proposed time set for calculating age standards is related to an almost equal proportion of patients from the old or pre-EU and EU admission eras, which accounts for their social differences and other influencing factors.

Crucially, our proposal to use different age standards for females and males is a significant analytical feature that may provide more meaningful insights than the exact year of their calculation. This distinction is particularly relevant when comparing cohorts of populations of similar age but with 2–4 decades of birth gaps. Gender-specific age standards can enhance the accuracy of health assessments and risk evaluations, as physiological differences between sexes can significantly influence aging and health outcomes. Future studies should consider the applicability of these standards across diverse populations to enhance their generalizability.

Our results are contextualized by several key studies utilizing AI for cardiac chamber volumetry, with methodological differences explaining variations in absolute values. Studies employing contrast-enhanced CT generally report higher absolute BVV values, a finding attributable to both the use of contrast and heart rate control. Shanbhag et al., using contrast-enhanced CT at 75% phase with heart rates controlled to ~60 bpm, reported a mean BVV of 384 mL in a cohort approximately a decade younger than ours [41]. The observed gradient from normal (350 mL) to overweight (387 mL) and obese (416 mL) individuals aligns with the physiological relationship between body size and cardiac volume. The higher absolute values in their study are likely multifactorial: the younger age of their cohort, the known effect of slower heart rate (increased by ~120 ms per cycle at 60 vs. ~68 bpm) to allow greater ventricular filling, and the potential volumetric effects of contrast enhancement itself [42]. This heart rate effect could account for a difference of approximately 30 mL.

In the domain of non-contrast cardiac CT, results vary based on the measurement technique and population. Daniel et al. reported a lower BVV of 237 ± 71 mL in a high-risk cohort from the MESA study [25]; however, this value was derived from 2D diameters and areas at a 50% acquisition phase, a method fundamentally different from our direct 3D volumetric approach. Conversely, a large validation study by Jacob et al. directly compared AI-based volumes from NCE-CT against CE-CT in a cohort of similar age (63 ± 13 years) but with a greater proportion of males (57%) [43]. Their recalculated mean BVV of 434 mL is substantially higher than our mean of 338 mL and is more comparable to values we observed in overweight and obese males. This discrepancy may be partly explained by their use of contrast-enhanced CCTA as the reference standard, potentially biasing their NCE-CT algorithm towards larger volumes.

Most compelling are the results from studies that closely mirror our NCE-CT methodology and clinical context. In a study conducted with 169 paired cases of the same individuals aged 62 ± 10 years, 53% females, who underwent CT lung cancer screening, the volumes of heart chambers and LV mass were determined by an AI-enabled volumetry [36]. The study showed that the BVV could be calculated from the NCE-CCT and chest NCE-CT images. Interestingly, the average AI-BVV was approximately 356 mL, which is very close to our overall finding [36]. Furthermore, the extension of the MESA project utilizing AI on CAC scans provided data for recalculating mean BVV, counting 345 mL in 5750 participants, which aligned with volumes in our normal and overweight groups [44]. Notably, in the 256 patients who subsequently developed heart failure over 15 year period, the initial BVV was significantly larger (389 mL), mirroring our results in high-risk obese patients. Importantly, when these MESA THV values were indexed for BSA (~251–273 mL/m^2^), the results were not far from our population mean of 260 mL/m^2^. This consistency in indexed values across vastly different studies and measurement techniques underscores a critical point: while absolute volumes may vary due to protocol differences, the systematic error introduced by BSA-based indexation is a persistent and independent limitation, further validating the need for our proposed SHI framework.

The biventricular volume can be recalculated from data of AI-based determination of the total heart volume. In the validation study of Jacob et al. [35], cardiac chamber volumes and LV mass from NCE-CCT have been compared with data from CCTA in 420 participants (43% females) with a mean age of 63 ± 13 years. The mean recalculated BVV was 434 mL, and BVV/BSA was 220 mL/m^2^. These results are different from the corresponding means from our study (i.e., 338 mL and 176 mL/m^2^) and is rather comparable with results observed in overweight and obese males. The gender proportion (67% females in our study) might account for the differences.

We have noticed that an increase in the volume of the heart chambers is not necessarily the rule in obese people. In our study, this increase was more readily observed in patients with a normal BMI and a balanced height-to-weight relation. This finding is consistent with the phenomenon of limited heart size growth despite weight gain, described over 140 years ago by Müller, the anatomist, who found that heart mass increases proportionally to body weight until reaching 100 kg. Conversely, with further weight gain, the heart’s weight does not increase, and the heart-to-body weight ratio decreases [45]. This historical observation remains relevant. Results of the postmortem study on 27,645 cases indicated that in the underweight and the obese population, only a small proportion of cardiac size could be explained by BMI (R^2^ = 0.04 and R^2^ = 0.02, respectively) [46]. A second possible explanation for our observation is that with increasing body weight, concentric remodeling predominates over eccentric remodeling, so volume should not increase proportionally, and the left ventricular mass-to-volume ratio increases.

The interpretation of biventricular volume (BVV) requires a nuanced approach that acknowledges its dynamic relationship with age, sex, and body composition. It is essential to recognize that an increase in heart volume is not inherently pathological but can represent a physiological adaptation to increased workload, as observed in athletes and heavy workers, including those who are overweight or obese. This phenomenon is most prominent in younger individuals. Consequently, the larger BVV observed in our youngest age-stanine cohort may reflect the upper spectrum of a healthy phenotype. A significant interpretative challenge is the potential inclusion of individuals with an “athlete’s heart.” While we lack exhaustive data on physical activity, the well-documented, consistent decline in cardiac volumes with age across imaging modalities suggests this subgroup is not the primary driver of our observed age-related trends.

More critically, our data argue that clinical focus must expand beyond cardiomegaly to include the equally important phenotype of abnormally small ventricular volumes—a condition known as cardiac sarcopenia [9]. This is frequently identified in the elderly and may be multifactorial, arising from primary age-related decline or, as a “sedentary heart” secondary to chronic disuse (e.g., from musculoskeletal comorbidities like osteoporosis and frailty) or related to chronic illness (e.g., chronic heart failure, pulmonary diseases) [8]. This concept is corroborated by Ash et al. [47], who reported smaller ventricular volumes (279 ± 71 mL; 168 ± 39 mL/m^2^) in patients with asthma and found this metric predicted higher exacerbation rates. The striking similarity of our findings in women with normal BMI (279 ± 62 mL; 170 ± 36 mL/m^2^) to these pathologically small values is alarming. It suggests that standards might misclassify a portion of the elderly population, masking a potentially critical sign of impaired cardiac reserve. To mitigate the risk of misclassification, a comprehensive clinical assessment, incorporation of additional imaging modalities, consideration of comorbidities, and longitudinal studies are essential to distinguish normal aging from pathology. Indeed, at a senile age, typical CV risk factors might play a less important role. Simply assigning a prognostic value to typical cardiovascular risk indicators from the 45–70 age category may be misleading and less meaningful in the older population, especially for women. This is simply a biological trap. In this context, the Charlson Comorbidity Index [48] might be a better tool than traditional CV risk scores for assessing the health status of the older population.

Interestingly, in alignment with the work of Juneau et al. [39], we did not observe significant differences in volumetric parameters based solely on the presence or absence of a conventional cardiovascular risk profile (e.g., hypertension, diabetes, subclinical CAD). This supports the notion that established risk factors, while prognostic for events, may not directly manifest as overt changes in cardiac chamber size in a cross-sectional analysis, further underscoring the need for sophisticated, multidimensional phenotyping tools like the SHI [49].

## 5. Conclusions

Collectively, these findings reveal a critical flaw in current clinical practice: the reliance on monolithic, universal reference values. Applying criteria derived from younger, athletic populations to older adults risks missing cardiac sarcopenia. Conversely, applying standards from older cohorts to younger individuals pathologizes normal physiological adaptation. Therefore, the use of age-stanines, sex-specific, and body habitus-adjusted is not merely beneficial but essential. The prevailing model of obligatory, one-size-fits-all cut-off values is inadequately simplistic for nuanced clinical application. This evidence-based challenge to the status quo is a necessary step towards precision medicine. We advocate for the development of stratified normative criteria to ensure the clinical assessment of BVV accurately reflects deviation from the healthy state for a specific demographic, ultimately improving diagnostic accuracy and patient care. This would be possible with a deterministic factor that provides a valid measure for individuals. The SHI enhances personalized assessment, supports retrospective analyses, and integrates seamlessly into existing phenotyping systems—marking a pivotal advancement in the accurate, individualized evaluation of heart size.

## Figures and Tables

**Table 1:** Characteristics of the examined population.

Parameter	Mean (SD)/Proportion
Age (years)	63.6 ± 10.6
Sex (females, F/males, M)	1606/860
Risk factors (count)	1.7 (0–4)
Smoking	525 (21%)
Systemic arterial hypertension (SAH)	1867 (75%)
Hyperlipidemia (HL)	1327 (54)
Type-2 diabetes mellitus (t2DM)	498 (20%)
Body mass index (BMI, kg/m^2^)	28.9 ± 4.5
Normal	624 (25%)
Overweight	985 (40%)
Obese	857 (35%)
Coronary artery calcium score (CACS) > 0	1589 (64%)

**Table 2:** Anthropometric data concerning sex and BMI.

Sex	BMI Categories	Age		Height		BSA		SHI		*n*
		Mean	SD	Mean	SD	Mean	SD	Mean	SD	2466
W	Normal	62.7	10.3	1.622	0.059	1.647 ^¶^	0.110	1.597 ^¶^	0.068	451
	Overweight	65.7 ^#^	10.0	1.616	0.061	1.792 ^¶^	0.111	1.439 ^¶^	0.052	609
	Obesity	64.7 *	9.69	1.611 ^#^	0.058	1.990 ^¶^	0.150	1.231 ^¶^	0.110	546
M	Normal	62.7	12.0	1.744	0.068	1.859 ^¶^	0.128	1.803 ^¶^	0.060	173
	Overweight	62.3	11.2	1.752	0.064	2.031 ^¶^	0.123	1.648 ^¶^	0.058	376
	Obesity	61.1	10.9	1.747	0.069	2.230 ^¶^	0.166	1.440 ^¶^	0.112	311

Abbreviations: W—women, M—men, BMI—body mass index, BSA—body surface area (after Mosteller [32]), SHI—standard human index (unitless), *n*—number, SD—standard deviation, Statistical significance, Student’s two-sided *t*-test, vs. normal (W) * *p* < 0.01, # *p* < 0.001; vs other ¶ *p* < 0.001.

**Table 3:** The frequency of individuals as combination of BMI and BP categories.

Bodyprint	BMI	Women	Men	All
Weight dominance	Normal	57 (2.31)	150 (6.08)	207 (8.93)
	Overweight	491 (19.91)	165 (6.69)	656 (26.60)
	Obesity	546 (22.14)	45 (1.82)	591 (23.97)
Total		1094 (44.36)	360 (14.60)	1454 (58.96)
Height–weight balance	Normal	370 (15.00)	2 (0.08)	372 (15.80)
	Overweight	118 (4.79)	211 (8.56)	329 (13.34)
	Obesity	-	266 (10.79)	266 (10.79)
Total		488 (19.79)	479 (19.42)	967 (39.21)
Height dominance	Normal	24 (0.97)	21 (0.85)	45 (1.82)
	Overweight	-	-	-
	Obesity	-	-	-
Total (percent)		24 (0.97)	21 (0.85)	45 (1.82)
Column total (percent)		1606 (65.13)	860 (34.87)	2466 (100)

Abbreviations: BMI—body mass index, BP—bodyprint (a dimensionless regulatory part of SHI), where BP = h − BSA (after Mosteller [32]); the numbers in brackets show percentage of total.

**Table 4:** Biventricular volumes across sex and BMI categories.

Sex	BMI	BVV		BVV/h		BVV/BSA		BVV/SHI		*n*
		Mean	SD	Mean	SD	Mean	SD	Mean	SD	2466
F	Normal	279 ^¶^	62	172 ^¶^	37	170	36	175 ^¶^	39	451
	Overweight	290 ^¶^	59	179 ^¶^	35	162 #	32	201 ^¶^	42	609
	Obesity	321 ^¶^	71	199 ^¶^	43	161 #	32	265 ^¶^	77	546
M	Normal	370 ^¶^	80	212 ^¶^	43	199	39	205 ^¶^	50	173
	Overweight	411 ^¶^	89	234 ^¶^	48	202	41	250 ^¶^	61	376
	Obesity	442 ^¶^	106	252 ^¶^	57	197	42	310 ^¶^	104	311

Abbreviations: W—women, M—men, BMI—body mass index, BVV—biventricular volume, h—height, BSA—body surface area (after Mosteller [32]), SHI—standard human index, SD—standard deviation, *n*—number differences between BMI subgroups. Statistical significance, vs. normal # *p* < 0.001; vs other ¶ *p* < 0.001, Student’s two-sided *t*-test.

**Table 5:** Biventricular volumes across sex and bodyprint categories.

Sex	BP	BVV		BVV/h		BVV/BSA		BVV/SHI		*n*
		Mean	SD	Mean	SD	Mean	SD	Mean	SD	2466
F	W>	308 ^¶^	67	190 ^¶^	40	162 ^¶^	32	234 ^¶^	69	1094
	W~h	276	59	172	36	168	36	177	38	488
	h>	253	67	158	40	174	32	146 ^¶^	69	24
M	W>	399 ^¶^	93	229 ^¶^	51	201	39	276 ^¶^	100	360
	W~h	429	97	244	53	199	42	316	78	479
	h>	317 ^¶^	69	187 ^¶^	39	189	39	189 ^¶^	40	21

Abbreviations: F—females, M—males, BP—bodyprint (unitless), W>—weight predominance, W~h, weight–height balance, h>—height predominance. Statistical significance, vs. W~h ¶ *p* < 0.001, Student’s two-sided *t*-test.

**Table 6:** Biventricular volumes across sex and risk categories.

Sex	Female			Male		
Risk	Low (*n* = 241)			Low (*n* = 72)		
BMI	Normal	Overweight	Obesity	Normal	Overweight	Obesity
BVV	286 ± 67	305 ± 59	317 ± 65	396 ± 72	409 ± 100	456 ± 123
BVV/h	175 ± 39	186 ± 33	196 ± 39	224 ± 35	230 ± 53	257 ± 66
BVV/BSA	172 ± 39	168 ± 29	159 ± 31	209 ± 33	197 ± 45	203 ± 46
BVV/SHI	178 ± 41	209 ± 40	259 ± 67	219 ± 35	247 ± 60	312 ± 106
Risk	High (*n* = 1364)			High (*n* = 788)		
BMI	Normal	Overweight	Obesity	Normal	Overweight	Obesity
BVV	277 ± 59	287 ± 59 ^a^	321 ± 72	367 ± 81	412 ± 88	441 ± 105
BVV/h	171 ± 36	178 ± 36	199 ± 43	210 ± 44	235 ± 48	252 ± 57
BVV/BSA	169 ± 35	161 ± 30 ^a^	161 ± 42	198 ± 40	203 ± 40	197 ± 42
BVV/SHI	174 ± 38	200 ± 42	265 ± 78	204 ± 45	250 ± 54	310 ± 87

Abbreviations: Low-risk subgroup: CACS—coronary artery calcium score, CACS < 10, normal blood pressure (BP), non-diabetic; high-risk subgroup: CACS ≥ 10 and/or high BP and/or type 2 diabetes and/or smoker and high lipids or structural abnormalities. Means ± 1 standard deviation are present. ANOVA was used for comparisons of interaction between risk and BMI categories: F(8, 1702) = 0.506, *p* = 0.845 in males and F(8, 3192) = 0.662, *p* = 0.726 in females; Student’s two-sided *t*-test was used for high- vs low-risk comparisons, “a” *p* < 0.05.

**Table 7:** Multiple regression analysis of BVV regarding the denominators applied.

Parameter	BVV/SHI	BVV/BSA	BVV/h	BVV/w
Sex	**−0.42**	**−0.41**	**−0.46**	**−0.29**
Age	**−0.22**	**−0.19**	**−0.21**	**−0.29**
Smoking	**0.04**	0.01	−0.02	**0.04**
SAH	**0.14**	0.03	**0.097**	**−0.04**
High Lipids	0.002	0.013	0.008	0.016
T2DM	**0.095**	−0.03	0.03	−0.01
CACS qual	0.024	0.014	0.02	0.004
**Multiple R^2^**	**0.27**	**0.22**	**0.13**	**0.13**
F	136.5	107.6	**148.3**	56.2

Abbreviations: BVV—bi-ventricular volume, SHI—standard human index BSA—by surface area, h—height, w—weight, SAH—systemic arterial hypertension, T2DM—type 2 diabetes mellitus, CACS—coronary artery calcium score’ qualitative categories (CAC < 10 = 0, CAC 10 or more = 1), R^2^—coefficient of determination, F—determinant of significance due to variance. Significant values of coefficients are highlighted in bold.

## Data Availability

We have not asked the examined subjects to allow their data to be available, as the study started long before when such statements had not been required.
